# Gender representation in animal-related proverbs: Algerian vs. Jordanian Arabic

**DOI:** 10.3389/fsoc.2023.1145200

**Published:** 2023-06-08

**Authors:** Zoubida Madani, Nimer Abusalim, Mohammad Rayyan

**Affiliations:** ^1^Department of English Language and Literature, School of Foreign Languages, The University of Jordan, Amman, Jordan; ^2^Department of European Languages, School of Foreign Languages, The University of Jordan, Amman, Jordan

**Keywords:** gender, animal terms, positive-negative associations, Algerian Arabic, Jordanian Arabic

## Abstract

**Introduction:**

This study explores the connotative meanings in animal-related proverbs used to describe the behavior of men and women in Algerian and Jordanian societies.

**Methods:**

A questionnaire with 46 Algerian and 45 Jordanian animal-related proverbs was distributed to 30 native Arabic speakers enrolled at the University of Jordan. The analysis focused on adapted categories with a gender perspective, including inferiority, weakness, stupidity, ill-nature, objectification, ugliness, positivity, and shrewdness.

**Results:**

Both Algerian and Jordanian animal-related proverbs exhibited diverse connotative meanings. Women were predominantly associated with derogatory connotations in both languages, portraying characteristics such as weakness, stupidity, inferiority, cunningness, and trickery. Similar characteristics were present in descriptions of men, but women in Arab cultures were consistently depicted as subordinate and denigrated. Conversely, men were portrayed with authority, control, superiority, and strength over women. Additionally, positive depictions included animals like gazelles, peacocks, partridges, cats, and horses to symbolize the beauty of women. Men's positive characteristics, such as strength, courage, and superiority, were associated with horses, camels, and lions.

**Discussion:**

This study highlights the prevalent connotations in animal-related proverbs used to describe men and women in Algerian and Jordanian societies. It reveals derogatory portrayals of women, reinforcing their subordinate status, while men are depicted with authority and power. However, positive representations emerged, attributing beauty to women and highlighting admirable qualities in men. These findings shed light on the complex dynamics of gender portrayal within cultural proverbs, emphasizing the need for further examination of these linguistic expressions.

## 1. Introduction

Without language, ideas cannot be conveyed effectively, and even adequate information cannot be provided to others (Chomsky, 1986; Barajas, 2010; Pinker et al., 2019). Proverbs are one of the methods used by different cultures to deliver meaning and opinion through social interactions. Sibarani (2004) proposed that any combination of words or sentences, including proverbs, can indicate the perceptions and traits of the culture under consideration. As a result, the meaning conveyed by proverbs can define the specific nature of any culture, as it is closely associated with the culture of native speakers. Mieder (2004) found that proverbs contained a variety of artistic and metaphoric language that was used to describe and portray something or someone through comparison.

Animals have a significant impact on human life. Therefore, according to Kövecses (2003a), several human behaviors are grasped and traced through the use and embodiment of animal behaviors (Lakoff and Johnson, 1980; Kövecses, 2003a). The animal terms found in proverbs serve as an icon representing certain facets of human life. For example, Olateju (2005) found that animal terms have been used metaphorically to describe individuals. According to research in this field, animal metaphors are perceived primarily in terms of culture and context.

This study aims at investigating the connotative meanings of animal-related proverbs used to address and describe the behavior of women and men in Algerian and Jordanian societies, i.e., how men and women are represented in the Algerian and Jordanian animal-related proverbs. This study seeks to unveil both positive and negative meanings associated with both genders.

### 1.1. Research questions

This study endeavors to answer the following research questions:

1. What are the connotative meanings of animal-related proverbs used in Algerian and Jordanian societies?
2. What animal terms are used to conceptualize men and women negatively and positively in Algerian and Jordanian societies?

## 2. Literature review

### 2.1. Proverbs

Several scholars have offered to define the term proverb. Simplistically put, a proverb is a brief conventional assertion used to further some social goal or encapsulate people's experiences (Seitel, 1972; Mieder, 1993; Finnegan, 2012; Norrick, 2014). Animal terms are commonly used in human speech because they help communicate a wide range of emotions (Lawrence, 1993; Kellert, 1997). As a result, the animal world is among the source domains that offer a plethora of metaphorical expressions. The Great Chain of Being (GCB; Lakoff and Turner, 1989) attributes a higher order form to humans as opposed to animals. Hence, several studies show negative connotations attached to animal metaphors. However, it is worth noting that some animal metaphors or proverbs may also express positive connotations, such as “lion” for courage (Rodríguez, 2009). According to Kövecses (2003b), although these conceptual metaphors may be universal cross-culturally, the intended meaning and use of a specific animal term may vary depending on the linguistic and cultural background in question.

### 2.2. The Great Chain of Being

According to Lakoff and Turner (1989), the Great Chain of Being Metaphor is the best method for interpreting proverbs. This theory is derived from the “Generic is Specific” Metaphor, which allows people to choose a common general-level structure from specific schemas stored in their minds. As a result, the Great Chain of Being Metaphor Theory (GCMT) lays out multiple attributes and behaviors between the categories of various chains to facilitate understanding one domain in terms of another (Fu, 2008). In line with this, Lakoff and Turner (1989) define the GCMT as “an ensemble, something like a string quartet, in which there are four members with separate entities, but who play together so frequently that their identity as a group is more prominent than their identities as individuals” (p. 172).

### 2.3. Gender ideologies and possible theories

Many proverbial analyses and studies show how power and ideology are closely related. For instance, researchers such as Martínez Garrido (2001) and van Dijk (2001) have emphasized how power relations are frequently enforced through coercion, particularly regarding gender. The creation and application of proverbs may be a reflection of society's attitudes toward gender and the reinforcement of established power structures.

It is critical to recognize how ideology influences power dynamics in this situation. According to Fairclough (2003), ideologies represent specific features of the outside world and help build and perpetuate power, domination, and exploitative relationships. Thus, a society's dominant ideologies can influence how power is used and upheld, including in the context of gender relations. Studying proverbs can be a useful technique to understand the beliefs and power structures at work in a particular society. For example, proverbs that uphold gender norms might be a sign of patriarchal ideologies that support male supremacy and female subjugation. Similarly, a move toward a more progressive philosophy that aims to overthrow established power structures can also be seen in proverbs that question gender expectations and advocate for equality.

### 2.4. Review of related studies

“Representations of Women in Moroccan Arabic and Berber Proverbs” by Ennaji (2008) provides insight into the representation of women in traditional Moroccan proverbs. According to Ennaji, these proverbs were based on outdated gender stereotypes that portrayed women as helpless, inferior, and dependent on men. Additionally, Berber proverbs emphasized women's physical attributes and reproductive abilities more than Moroccan Arabic proverbs, frequently limiting women to domestic roles. Ennaji argues that using proverbs helped maintain gender inequality by reinforcing existing patriarchal standards. However, it was also noticed that some proverbs went against conventional gender roles, indicating some variation in how women were portrayed in Moroccan and Berber cultures. The study stresses the importance of examining linguistic and cultural artifacts to comprehend how gender is created and maintained in society.

Al-Harahsheh (2020) examined how Jordanian Spoken Arabic (JSA) used animal names metaphorically and vocatively to address people negatively or positively. A list of 44 animal names was distributed among 100 undergraduate students from Yarmouk University in Jordan as part of a survey. Participants had to complete a variety of tasks, including indicating whether they used animal names to refer to other boys or girls, surmising the implied meanings attached to these names, naming the grammatical structures in which they used these names, and providing instances of when they were used in Jordanian Spoken Arabic (JSA). The study concluded that Jordanians gave people animal names based on their demeanor, behavior, IQ, and character and that these animal names were primarily used as insults. Therefore, calling people by animal names was a linguistic and cultural phenomenon.

Several other studies have examined animal-related proverbs in different societies and cultures as tools to illustrate gender roles and stereotypes. Kuipers and Verdonk (2018) investigated how gender stereotypes were expressed in Dutch and Moroccan culture through animal-related proverbs. Both cultures had proverbs representing men as courageous, strong, and dominating while portraying women as feeble, sensitive, and submissive. The researchers concluded that in these cultures, the employment of proverbs reinforced conventional gender norms and stereotypes.

Likewise, Oduolowu and Adegoke (2020) examined using animal-related proverbs in Yoruba culture in Nigeria to depict gender stereotypes. According to the study, women were frequently characterized as nurturing, emotional, and subservient in Yoruba proverbs, while men were commonly portrayed as aggressive, strong, and domineering. The researchers concluded that the employment of proverbs promoted traditional gender norms and pushed women more to the margins of Yoruba society.

In South Korean culture, Kim and Park (2017) found that animal-related proverbs were frequently employed to convey gender norms and expectations. For instance, in the proverbs, men were often portrayed as exhibiting traits like power, bravery, and leadership and frequently likened to lions, bulls, and eagles. On the other hand, women in proverbs were often featured as doves, deer, and swans to represent traits like beauty, kindness, and nurturing. Their study also established that animal-related proverbs reinforced gender preconceptions and traditional gender norms in Korean society.

Similar findings were obtained in a study in the United States by Taylor et al. (2018) which analyzed a sample of 200 proverbs. The study found that animal-related proverbs were frequently employed to explain gender differences and expectations. Proverbs related to men often portrayed traits like power, aggression, and dominance and frequently used animals like lions, wolves, and bulls for men. In contrast, proverbs about women often portrayed traits like beauty, passivity, and emotional sensitivity and frequently used butterflies, swans, and doves for women. The study concluded that gender prejudices and inequities in American culture were reflected through these animal-related proverbs.

Khan et al. (2017) investigated the portrayal of men and women in Urdu proverbs through animal terms. Approximately 40 Urdu proverbs with animal metaphors were collected, and their categories of analysis were studied through a gender lens. The selected animal proverbs were analyzed and classified into categories such as inferiority, weakness, stupidity, ill-nature, sex object, ugliness, positive, and shrewd.

In these proverbs, dogs were portrayed as inferior, and men were regarded as superior. Comparing a man to a dog was deemed abusive in Pakistani society; however, in the sample proverb where the dog was linked with man, the connotation was negative. Similarly, the monkey symbolized ugliness and inferiority and was used as an insult. On the other hand, the cat was comparable to the traditional domestic role of women; for instance, “women cannot sedate men, just as a cat cannot teach a lion.” Women were also considered difficult to understand and were presented as a riddle in some Urdu proverbs.

The cow and buffalo have frequently been used as metaphors for women. In traditional societies, women were seen as slow, stupid, dumb, and obedient like cows. Conversely, men were influential in a patriarchal society and owned women by force. Men were likened to the camel, which is a symbol of strength. The camel symbolized male dominance in Pakistani society and helped establish societal norms. The hen was used as a metaphor for women and portrayed them as weak and inferior compared to how men were described in the proverbs. Men's voices, rules, and dominant roles were accepted by society. The snake is generally regarded as a dangerous animal because of its poison, and represents evil. It was more frequently associated with men in Pakistani society, emphasizing negative representations of men.

Adopting a feminist critical approach, Aragbuwa and Omotunde (2022) employed linguistic frameworks to investigate conceptual metaphorizations in gender-based Yoruba proverbs. The data set, containing 100 Yoruba proverbs about women, was used for the Conceptual Metaphor Theory and Feminist Critical Discourse Analysis. The analysis revealed that women were structured in the following four conceptual metaphors in the selected proverbs: women as weaklings, women as evil, women as whores, and women as procreants. While the first three conceptual metaphors explicitly suggested women in a “downward orientation,” the fourth metaphor suggested an “upward orientation,” though, in reality, it implied a downward orientation. The overall negative image of these four metaphors indicated that the status of women was poor among the Yoruba. Yoruba's ideological gender structure promoted a hierarchical order where women were subordinate to men. As a result, the systematic use of derogatory language to portray women among the Yoruba exposed their (mis)conception of women. The study highlighted how the Yoruba used conceptual metaphors to express their gender relations, and positive characteristics of women that contradicted these conceptual metaphors were masked.

The reviewed literature shows that gender-based analyses of animal-related proverbs have been conducted in different languages. Still, these studies primarily focused on the image of women in different languages. Very few studies have compared the image of men and women in society through these proverbs. Therefore, this study attempts to fill this gap by investigating how the Algerian and Jordanian societies use animal-related proverbs when portraying men and women.

## 3. Methodology

### 3.1. Participants

The participants included 30 graduate students from the University of Jordan, Algerian (*n* = 15) and Jordanian (*n* = 15). It was convenient to locate native speakers of Algerian Arabic among the Algerian graduate students, as the primary researcher is a graduate student at the University of Jordan. Similarly, graduate students speaking Jordanian Arabic were also selected. The respondents were natives of their respective dialects, i.e., Algerian and Jordanian Arabic.

### 3.2. Instrument for quantitative analysis

To answer the research questions and meet the study's aims, the researchers designed a questionnaire containing 46 Algerian and 45 Jordanian animal-related proverbs. The questionnaire was then distributed to the respondents. It comprised two parts; the first part had ethnographic data relating to the participant's age, gender, and nationality. The second part included animal-related proverbs against which participants had to indicate whether a certain proverb related to men, women, or both and whether they connoted a positive or negative meaning.

### 3.3. Analytical framework for qualitative analysis

Khan et al.'s (2017) classifications were modified and extended for an in-depth linguistic and gender investigation of the chosen proverbs. In their study, they used categories such as inferiority, weakness, stupidity, ill-nature, sex object, ugliness, positive, and shrewd. To keep the analysis neutral, counter-classifications for the categories listed in their study were created (Khan et al., 2017). The following are the categories adapted from Khan et al. (2017).

#### 3.3.1. Inferiority vs. superiority

In Khan et al.'s (2017) study, the classification of animal terms under this category was motivated by the Urdu culture's view of the animal “dog,” in particular, as an inferior animal. In addition, the aspect of power is emphasized to show the dog's dominance over its area. Conversely, the dog is powerless outside its surroundings. Worthlessness, obedience, and ignorance are other traits highlighted under this category. In some examples provided by the authors, women were likened to animals that were inferior in classification compared to those used for men. This was justified by the power and control men had over women. For instance, Khan et al. (2017) provide examples of the cat's inferiority and the lion's superiority by depicting the cat as a domestic, powerless animal that should not aspire to tutor the lion.

#### 3.3.2. Weakness vs. strength

Strength and power are two main characteristics in this category and are mainly linked to male animals. In addition, all aspects of physical strength/weakness, shape, and size are also included under this category. Khan et al. (2017) suggest that the camel is a dangerous animal compared to the dog, based on an Urdu proverb that urges people to be cautious of the grip of a camel and the deception of women. The authors explain that even though the dog is dangerous, it is still not envious like the camel. Regarding body shape and strength, the authors use the buffalo to show that a stick can take such a huge animal.

#### 3.3.3. Stupidity vs. wisdom

Under this category, the authors highlight that the size of an animal is not necessarily a symbol or a sign of intelligence. Further, stupidity was associated mainly with donkeys in this section, as it is a slow, obedient, easily manipulated, and good-natured animal. As a means of exemplification, the dog is compared to the donkey, and the results indicate that the dog is an intelligent creature compared to the donkey.

#### 3.3.4. Ill-nature vs. good nature

Unlike the category of strength and weakness, which focuses on male animals, this category primarily focuses on female animals. This is exemplified by the wasp, a flying insect that can be a source of danger if its hive is disturbed. Other characteristics under this section include bad temper, manipulation, aggressiveness, and evil. The authors portray the snake as an ill-natured animal not just because of its poison but also due to its vile nature. In Islamic belief, the snake is believed to be responsible for the condemnation of Adam and Eve.

#### 3.3.5. Sex object vs. authority

This category associates women's beauty, sensitivity, and delicacy with the feature of sex objects. However, men are associated with the term authority since men are known for their power, strength, and stronger, more rugged bodies. Therefore, the Algerian and Jordanian proverbs discussed under this category reflect either one of these features, i.e., sex object or authority or both. The animals themselves are not presented as sex objects. However, their characteristics are linked to the respective genders.

#### 3.3.6. Ugliness vs. beauty

This category focuses on aesthetic appearance, and women, their youth, and elegance are at the center of this beauty classification. However, in Khan et al.'s (2017) study, ugliness was chiefly linked with men, and they supported this with an example of the animal “monkey.” In addition, when horse and donkey are compared, the horse takes the lead over the donkey as it is considered a beautiful animal, unlike its counterpart, which is perceived as a lowly and ugly animal.

#### 3.3.7. Positive vs. negative

The negative feature of this category relates to the idea that if people or animals have bad habits and attitudes, they will, by one means or another, affect others surrounding them. According to Khan et al. (2017), dog and fish connote negative meanings. Almost all female animals are pictured in a negative light. Contrary to this, the hen symbolizes a positive meaning as it is a precious and valuable creature that two roosters cannot share. This applies to women, mainly in the Islamic and Arabic cultures. Being useless and having poor control over a given situation is also considered a negative characteristic.

#### 3.3.8. Shrewd vs. foolish/innocent

According to Khan et al.'s (2017) classification, the term shrewd represents an artful, cunning, and tricky nature. They further suggest that the term “innocent” could be used as an opposite feature to shrewd. In their study, the camel and the cat are shrewd animals since the camel's sitting direction cannot be predicted, and the cat does cunning acts that cannot be forgiven. These attributes are equally applicable to human beings in the proverbs.

The listed classifications have been used to depict both genders using animal metaphors for positive and negative features. The selected proverbs were categorized and investigated in these categories, and their gender portrayal was examined through animals associated with them.

### 3.4. Procedures

The current study used a mixed methodology consisting of qualitative and quantitative analyses. The qualitative data came from the connotative meanings of the animal-related proverbs (suggested by the participants and the researchers' native perceptions) and the gender these proverbs were linked with (men or women). In addition, the qualitative data also included characteristics that these proverbs ascribed to a man or a woman (such as physical appearances and behaviors). This was in line with the framework adopted in this study from Khan et al.'s (2017) categories of analysis through a gender perspective, which helped unveil whether these animal-related proverbs were used derogatorily or complimentarily for both genders. The qualitative data also helped determine if men and women were described using the same animal terms or whether there were discrepancies.

The quantitative data helped establish the frequency of each animal term used in this study (if more than one proverb contained the same animal name, then the animal name was counted that many times). Positive and negative occurrences were also counted. In addition, the proportion of animal terms used exclusively to describe women or men was calculated to examine whether the same animals were used for both genders to represent each category or whether some animals were not used to describe men or women. The tables pertaining to the quantitative analysis were separated based on dialect.

After collecting the data, the proverbs were divided into two: Algerian animal-related proverbs and Jordanian animal-related proverbs. The data was then tabulated, translated, transliterated, and a possible explanation for each animal-related proverb was provided based on qualitative data gathered from the participants, the primary researcher's insight into the Algerian data, and the secondary researcher's insight into Jordanian data. In addition, the tables also contained responses from men and women from Algerian and Jordanian society, along with the positive and negative connotations of the proverbs under scrutiny.

## 4. Results

In this section, the results of the Algerian and Jordanian Arabic proverbs have been presented and discussed. Section 4.1 presents the quantitative results, and Section 4.2 offers the qualitative analysis. Since the focus is on connecting the animal term to the frequency of its use instead of connecting the proverb to such use, each subcategory (animal term) has been indicated with a number in parenthesis which shows the number of instances it appeared in the list of proverbs under investigation. For instance, in Table 1, the animal term “donkey” was found in six proverbs and has been indicated with a number six against its term. The term had negative connotations in all six proverbs and was never used in a proverb to create a positive connotation. Further, “donkey” was used in three proverbs to describe women and three proverbs to describe men.

**Table 1:** Percentage of Algerian animal terms.

**Animal term**	**Algerian data**							
	**Positive**		**Negative**		**Men**		**Women**	
	**Frequency**	**%**	**Frequency**	**%**	**Frequency**	**%**	**Frequency**	**%**
Donkey (6)	00	00	6	13.04%	3	50%	3	50%
Dog (6)	00	00	6	13.04%	4	66.67%	2	33.33%
Cow (2)	00	00	2	4.34%	1	50%	1	50%
Horse (4)	3	6.52%	1	2.17%	1	25%	3	75%
Sheep/goat (3)	00	00	3	6.52%	00	00	3	100%
Cock (3)	1	2.17%	2	4.34%	2	66.67%	1	33.33%
Worm (1)	00	00	1	2.17%	00	00	1	100%
Rat (2)	00	00	2	4.34%	1	50%	1	50%
Snake (4)	00	00	4	6.52%	2	50%	2	50%
Bee (1)	00	00	1	2.17%	00	00	1	100%
Wolf (3)	00	00	3	6.52%	2	66.67%	1	33.33%
Duck (1)	00	00	1	2.17%	00	00	1	100%
Cat (1)	1	2.17%	00	00	00	00	1	100%
Beetle (3)	00	00	3	6.52%	1	33.33%	2	66.67%
Gazelle (2)	1	2.17%	1	2.17%	1	50%	1	50%
Camel (4)	1	2.17%	1	2.17%	1	50%	1	50%
Lion (1)	1	2.17%	00	00	1	100%	00	00
Peacock (1)	1	2.17%	00	00	00	00	1	100%
Partridge (1)	1	2.17%	00	00	00	00	1	100%
Bird (2)	1	2.17%	1	2.17%	2	100%	00	00
Monkey (1)	00	00	1	2.17%	1	100%	00	00
Fish (1)	00	00	1	2.17%	1	100%	00	00
Fly (1)	00	00	1	2.17%	1	100%	00	00

### 4.1. Quantitative analysis

Table 1 displays the frequencies and percentages of each animal term used and the percentage of their intended positive and negative meanings. Of the 23 animal terms employed in 46 Algerian animal-related proverbs, the most used animals were donkeys, horses, dogs, wolves, camels, snakes, sheep, and beetle, followed by cats, ducks, worms, birds, fish, flies, lions, partridges, and peacocks. In some instances, animal terms described only men (i.e., bird, lion, monkey, wolf, fly, and fish), whereas in other cases, it represented only women (cat, duck, bee, snake, and worm).

It was also observed that negative connotations exceeded the positive ones for both genders (Table 1).

Table 2 shows the data obtained from the Jordanian animal-related proverbs, representing 19 animal terms used in 45 proverbs. The table indicates the use of some animal terms that were not used in the Algerian proverbs. For instance, crow, hen, ant, and louse are only found in the Jordanian data. The most used animal terms include sheep, horse, camel, wolf, snake, donkey, and dog, followed by monkey, rat, and lion.

**Table 2:** Percentages of Jordanian animal terms.

**Animal term**	**Jordanian data**							
	**Positive**		**Negative**		**Men**		**Women**	
	**Frequency**	**%**	**Frequency**	**%**	**Frequency**	**%**	**Frequency**	**%**
Dog (3)	00	00	3	6.67%	3	66.67%	1	33.33%
Sheep/goat (6)	00	00	6	13.33%	4	66.67%	2	33.33%
Louse/louse eggs (2)	00	00	2	4.44%	00	00	2	100%
Cow (1)	00	00	1	2.22%	00	00	1	100%
Hen (1)	00	00	1	2.22%	00	00	1	100%
Horse (4)	4	8.88%	00	00	1	25%	3	75%
Donkey (3)	00	00	3	6.67%	2	66.67%	1	33.33%
Camel (8)	2	4.44%	6	13.33%	4	50%	4	50%
Snake (3)	00	00	3	6.67%	1	33.33%	2	66.67%
Rat (3)	1	2.22%	2	4.44%	1	33.33%	2	66.67%
Bird (1)	00	00	1	2.22%	1	100%	00	00
Wolf (4)	1	2.22%	3	6.67%	4	100%	00	00
Ant (1)	00	00%	1	2.22%	1	100%	00	00
Crow (1)	00	00	1	2.22%	1	100%	00	00
Gazelle (1)	00	00	1	2.22%	1	100%	00	00
Monkey (2)	00	00	2	4.44%	2	100%	00	00
Lion (2)	1	2.22%	1	2.22%	2	100%	00	00
Hyena (1)	1		00	00	1	100%	00	00
Beetle (1)	00	00	1	2.22%	1	100%	00	00

Notably, pejorative connotations were more than positive references and even more than those found in the Algerian data. Some overlap was evident between what certain animals represented regardless of culture; they are universally seen as positive/negative. However, the results also indicated that some animal terms used in describing people might be culture-specific (for instance, crow, hen, ant, and louse in Jordanian Arabic).

### 4.2. Qualitative analysis

This section analyzes how men and women from Algerian and Jordanian societies are represented in animal-related proverbs. The positive and negative meanings are discussed, along with the classification of the proverbs according to the framework adopted for this study.

#### 4.2.1. Inferiority vs. superiority

The category of inferiority vs. superiority relates to the ranking and positioning of women in society with regard to men; it signifies whether Algerian women are perceived as inferior or superior compared to Algerian men.

From the proverb (1) (اللّي عينو في لعذاب يخالط النسا و لكلاب), “*The one who wants suffering should mingle with the women and the dogs*,” it is deduced that women are likened to dogs. Although the dog represents loyalty in almost all Western cultures and the Arab world, it is considered unclean (nadʒis/dirty) among Muslims. In this proverb, by equating women's wailing to dogs barking, women first are derogated and second are portrayed as a source of suffering. It implies that if a man wants to suffer and place himself in trouble, he only needs to get in touch with women and dogs, both loud and noisy. It is also possible that the proverb suggested women embodying moodiness due to their constant wailing.

#### 4.2.2. Physical strength vs. weakness

The second category relates to physical strength vs. weakness in animal terms. Proverbs in this category either compare and contrast women as weak and men as strong or just portray women as weak in animal terms. In proverb (2) (النسا اذا تحزمت والخيل اذا تلجمت), “*Women if they gird and horses when harnessed*” equates women to horses in their strength; once they are ready (saddled), they are ready for battle. Likewise, women encircling their garments around their waist suggests their preparedness to clean the house or cook. This imagery in the Algerian culture reflects the suaveness and sharpness of the Algerian women, who, by doing so, are regarded as good housewives.

#### 4.2.3. Stupidity vs. wisdom

The stupidity vs. wisdom category included examples of how the good nature of some animals was manipulated by others. This characteristic was applied to human beings, where a man or a woman uses someone, mocks them, and takes advantage of their kindness.

Proverb (3) (ما تسمع رأي المرا ما تّبّع الحمار من وراء) “*Do not listen to the point of view of the woman and do not follow the donkey from the back,”* advises on how a woman's point of view is worthless. The proverb likened women to a donkey, where following it from behind will either result in getting kicked by it or getting dirty by its dung. It suggested that following a woman, with their inability to judge with reason and lack of experience, will result in taking the wrong path or making the wrong decision.

#### 4.2.4. Ill-nature vs. good nature

Animals, like humans, have both good and bad qualities. Ill-nature can be defined as being short-tempered, manipulative, aggressive, or evil.

In this category, five proverbs describe women as deceptive, cunning, witty, and malicious. The fourth proverb (طبيبة اللّفعة تولّي حبيبة الربيبة تولّي ملّي), “*When the stepdaughter becomes deer, the snake becomes a doctor*,” underscores the relationship between the stepdaughter and the stepmother. It is common in the Algerian community for a man to remarry after his wife's death. If he already had children, they would not always like the stepmother. Therefore, this proverb mocks any possibility of a good relationship between the stepmother and the step-children; it is as remote as the snake becoming a doctor. The snake is poisonous and known for hissing and striking people, which is genetic and innate and cannot be changed. The impossibility of a good relationship between the stepmother and the stepchildren is likened to the character of a snake, which cannot change.

#### 4.2.5. Sex object vs. authority

In the proverbs, women are portrayed as sex objects and personifications of gorgeousness and delicacy. The opposite of this quality is authority, which is a defining feature for men and frequently described as such in proverbs. In the proverb **(**وظرافة خفّة حنّة، ظبيّة فلانة**)** “*She is a female antelope; henna, lightness and cuteness*,” women are seen showing off and projecting their beauty. That is especially true when a mother wants to praise her daughters and show that they are beautiful in form and content, then they depict them as a female antelope known for its lightness and beauty. In addition, women are also seen to represent such beauty as something pure like henna, which is glorified and preserved in bags made of doe leather.

#### 4.2.6. Ugliness vs. beauty

Beauty is usually linked with women, especially their physical appearances. Even among animals, beauty is expressed in physical traits such as the gazelles' eyes or the horse's nobility, youth, and magnanimity. These traits can be likened to human behaviors and physical appearances. However, beauty in this context also describes the criteria for the price and job animals do. In proverb (6), (بطّة صحاحت وايلا قطة ضعافت ايلا لقصيرة لمرا) “*The short woman, if she is skinny, she is a cat, and if she is fat, she is a duck*” describes the beauty of a woman as a cat, if she is slim. The woman is likened to the cat's fitness and agility. However, when a woman gains weight, she is described as a duck because of her way of walking and body shape. The duck sways and is puffy; likewise, the woman will automatically sway while walking after gaining weight.

A brief overview of the proverbs indicates that the negative meaning used to describe women are more than the positive ones. Moreover, the derogatory image of women is conceptualized using these animal terms; mule, dog, cow, sheep, cock, worm, donkey, rat, snake, wolf duck, beetle, scorpion, and camel (see Table 3). The positively used animal terms are horse, cat, deer, gazelle, peacock, and partridge. The results reveal that the animal imagery of women in Algerian society is pejorative in its nature. These results align with Ennaji's (2008) study, where the animal terms such as beetle, sheep, dog, donkey, cow, and snake are negatively used to describe women.

**Table 3:** Algerian animal-related proverbs describing women.

**Categories**	**Women animal-related proverbs along with their translation**	**Positive meaning**	**Negative meaning**
1. Inferiority vs. superiority	اللّيعينوفيلعذابيخالطالنساولكلابʔilli ʕaynu: fi leʕða:b jxa:latʕ ʔinsa wa lakla:b **Transliteration:** The one who wants suffering should mingle with the women and the dogs. **Possible translation:** Those who want torment shall get in touch with women and dogs. **Meaning:** If you want to have a headache you just need to have women and dogs. Because a woman's wailing is like dogs' barking. In this example, the woman's situation is inferior in nature since it is compared to a lowly classified animal.		-
2. Weakness vs. strength	النسااذاتحزمتوالخيلاذاتلجمتʔansa ʔiða tħazmet wa lxa:jl ʔiða tledʒmat **Transliteration:** Women if they gird and horses when harnessed. **Possible translation:** The women if they gird are like horses if they are harnessed. **Meaning:** When the woman gets ready for household things, she is like the horse being prepared and decorated by its saddle. This example depicts the power of a woman even if she is a weak and sensitive creature, but when needed she becomes as powerful as a horse.	+	
3. Stupidity vs. wisdom	ماتسمعرأيالمرامااتّبّعالحمارمنوراء ma tesmaʕ ra:j lemra ma ʔəttabeʕ leħma:r man wra **Transliteration:** Do not listen to the point of view of the woman and do not follow the donkey from the back. **Possible translation:** Do not listen to the woman's point of view nor follow the donkey from the back. **Meaning:** Do not allow a woman to take the lead nor dare you walk behind the donkey because it may kick you. Thus, women and donkeys are not worth taking the lead. This example shows that a man should not be stupid by doing what is denied in the proverb. In addition, based on Khan et al.'s (2017) classification the donkey is a symbol of slowness and stupidity.		-
4. Ill-nature vs. good nature	ملّيتولّيالربيبةحبيبةتولّياللّفعةطبيبة malli twalli ʔirbi:ba ħbi:ba twalli ʔallafʕa tʕbi:ba **Transliteration:** When the stepdaughter becomes deer, the snake becomes a doctor. **Possible translation:** When the stepdaughter becomes lovely and sweet in the eyes of the stepmother is when the snake will become a doctor. **Meaning:** This proverb is a mockery and shows that having a good relationship between the stepdaughter and the stepmother is something impossible just like when a miracle happens and turns the snake into a doctor. This example shows that the snake is an evil animal and it is impossible for it to let down its characteristics of vile and bad temperedness. Therefore, the stepdaughter cannot be a good person because this is in its nature.		-
5. Sex object vs. authority	فلانةظبيّةحنّة،خفّةوظرافة fla:na ðʕabijja ħanna xaffa wa ðʕra:fa **Transliteration**: She is a female antelope; henna, lightness and cuteness. **Possible translation**: She is like a female antelope, henna, lightness and cuteness. **Meaning**: This proverb depicts what women say to be proud or to glorify their daughters that they are beautiful in form and content, and there is nothing better and purer than preserved henna in bags made of doe leather. This proverb fits the characteristic stated by Khan et al. (2017) in this category; that is, sex object is linked mainly with women's delicacy and softness.		-
6. Ugliness vs. beauty	لمرالقصيرةايلاضعافتقطةوايلاصحاحتبطّة lemra laqsʕi:ra ʔila dʕʕafet qatʕtʕa wʔila sħa: ħet batʕtʕa **Transliteration:** The short woman, if she is skinny she is a cat and if she is fat she is a duck. **Possible translation:** If the short woman loses weight she is like a cat, but if she gains weight she is like a duck. **Meaning:** The woman is likened to the cat's fitness and agility. However, when a woman gains weight she is described as a duck in her way of walking and her body shape. This proverb shows both sides of this category; the first one depicts the positive side and beauty of a woman as having a light body like a cat which allows it to move easily. However, the second part depicts the ugly side of a woman when she is fat; as her body shape will not be as beautiful as when she is skinny.	**+**	-
7. Positive vs. negative	فلانةكيالحمارةالرّادفماتصكماتفك fulana ki leħmar ʔerradef ma tsʕukma tfu:k **Transliteration:** She is like a pregnant donkey; she does not kick nor solve problems. **Possible translation**: She is like the pregnant donkey that can neither kick nor help in resolving a dispute. **Meaning**: The woman that is useless does nothing beneficial for her family nor her society. According to Khan et al.'s (2017) classification, almost all female animals connote a negative meaning and since being lazy is a bad habit, this also can be an aspect affecting others surrounding her.		-

Table 3 lists the Algerian animal-related proverbs describing men. Proverb (8) (الوقت تقلّب ولحمار ولّى على العود يجلّب) “*The time turned, and the donkey is jumping over the horse*,” belonging to the first category, describes a situation when time changes and the norms are inverted. In this example, the horse represents someone strong and superior to the donkey, which is perceived as lower than the horse and cannot rival it. The proverb indicates a time when an unfit person assumes a prestigious position in the place of a more deserving good man. This is portrayed as a donkey jumping over a precious horse.

Proverb (9) (الجمل ما يطابس على حمصة) “*The camel does not bow to a chickpea*,” talks of the camel, known for its strength, stamina, patience, and height. The proverb uses this image to describe a man who does not sweat over small things. In other words, a man of good standing is not preoccupied with small things that have no benefit and may lessen his position and merit. From proverbs such as these, it can be deduced that the Algerian society portrays men positively by likening them to strong animals such as the camel.

However, proverb (10), belonging to the third category, does not project men favorably. The proverb (يدور اللّيل في و يبور النهار في الدّرسة بغل كي يجعلك الله) “*May Allah make you like a donkey turning in the day and a bachelor at night*,” provides a derogatory image of man by characterizing him as a mule, which is considered a stupid animal. Through this proverb, Algerian society compares the life of a married man with that of a bachelor. After a tiring day, the married man will return to his warm house, wife, and children. However, the bachelor works very hard during the day (like a mule), but at the end of the day, he has no one to entertain him.

Proverb (11) (حوتة ومطليّة بالزّيت) “*A fish covered in oil*” describes a fish that has become more slippery and hard to catch because it has been sprayed with oil. This proverb is used to express some people characterized by their cunningness and wit. When a person's behavior or act cannot be predicted, this proverb is used to reveal the person's foxy and malicious traits. The proverb warns people to be cautious when dealing with such persons because they are like oiled, slippery fish that cannot be caught.

Proverb (12) (كي غلبوه الديوكة ولّى على موكة) “*When the cocks beat him, he turns back on Mouka (the hen)”* suggests that when roosters attack another rooster, the latter will show off and demonstrate his power and strength on another, weaker bird, mainly the hen, referred to in the proverb as Mouka. The proverb describes a man with no authority among his friends and whose word is worthless. Yet, he will return home and boast his abilities to his wife, who is in a lower position than the man and has no power to challenge him. The proverb also reaffirms the authority men have over women.

On ugliness and beauty, proverb (13) (يرد يشمها كي الفايح للكلب الروايح دير) “*Put the perfumes on the stinky dog, and when it smells it, it denies it”* suggests that despite all benevolence attributed to a dog, at the end of the day, it will still remain a dog, just as a leopard cannot change its spots. This proverb also indicates a crude person who cannot distinguish between valuable and cheap things.

The proverbs referring to the characteristics, behaviors, and physical appearances of Algerian men show almost the exact usage of animal terms found in the proverbs describing women, projecting them favorably or disadvantageously to men in Algerian society. The following animal terms were used to project a positive image of men: horse, camel, lion, bird, and cock. On the other hand, dogs, donkeys, ants, wolves, bulls, fish, snakes, rats, beetles, monkeys, and hyenas were used to portray men negatively. While comparing men and women, the superiority of men reigned supreme. This was evident in the use of the animal pair cock-hen, which was similar to that used in Jayawardena's (2015) study, which investigated how women were portrayed in two cultures—Sinhala and French. The study used the animal pair cock-hen to describe women's obedience toward their husbands and men's authority over women; this behavior was equated to that one exercised by the cock.

**Table 5** lists the Jordanian animal-related proverbs describing women in their society. Interestingly, the category positive vs. negative used to describe Algerian women (see Table 4) was missing from the Jordanian proverbs of women; instead, we found proverbs belonging to the shrewd vs. innocent category.

**Table 4:** Algerian animal-related proverbs describing men.

**Categories**	**Animal-related proverbs along with their translation and meaning**	**Positive meaning**	**Negative meaning**
8. Inferiority vs. superiority	الوقتتقلّبولحمارولّىعلىالعوديجلّبʔalwa:qt tqallab wleħma:r walla ʕla lʕawd ʔidʒellab **Transliteration:** The time turned and the donkey is jumping over the horse. **Possible translation:** The times have changed and the donkey now wants to surpass the horse. **Meaning:** Someone lower in position wants to overpass a highly ranked one. Since the donkey is perceived as an obedient and easily manipulative animal, then it is lower in position and importance as compared to the horse. The latter, is a noble animal and it is not an easy task to tame it.		-
9. Strength vs. weakness	الجملمايطابسعلىحمصة ledʒmal ma jtʕa:bes ʕla ħu:msʕa **Transliteration:** The camel does not bow to a chickpea. **Possible translation:** The camel does not bow to get a piece of chickpea. **Meaning:** The strength of a camel is likened to a strong, noble and gentle man who does not bother himself with silly things and does not allow for silly people to underestimate him. As in the study of Khan et al. (2017), the camel symbolizes physical strength. The same applies in this example; as to show the greatness of the camel or a powerful person.	+	
10. Stupidity vs. wisdom	اللهيجعلككيبغلالدّرسةفيالنهاريدوروفياللّيليبورʔalla:h jadʒaʕlek ki bɤa:l ʔeddarsa fi nha:r jdu:r w filli:l jbu:r **Transliteration:** May Allah make you like a donkey turning in the day and a bachelor at night. **Possible translation:** May Allah make you like a plowing mule that keeps cultivating all the day and sleeps alone at night. **Meaning:** The donkey has nothing to do, it is a symbol of stupidity and all the day it just keeps turning and cultivating. Therefore, a stupid person will do the same having no purpose or goal to achieve so he will keep turning around and may even not be able to have a family and get established.		-
11. Ill-nature vs. good nature	حوتةومطليّةبالزّيتħu:ta wmatʕlijja bezzi:t **Transliteration**: A fish covered in oil. **Possible translation:** Like a fish coated by oil. **Meaning:** The man is so slick and shrewd that he cannot be predictable. The bad and evil person can have the ability to hide his true intents and can find a way out of any trouble or issue.		-
12. Sex object vs. authority	كيغلبوهالديوكةولّىعلىموكة ki: ɤalbu:h ʔedju:ka: wella ʕla mu:ka **Transliteration:** When the cocks beat him he turns back on Mouka. **Possible translation:** When the roosters defeated him, he prevailed over Mouka (the hen). **Meaning:** This proverb depicts the male who is defeated in front of his peers and competitors, and then he transgresses against someone who is weaker than him because of his villainy. That is to say, this is an instance of the man's authority over his weak wife.		-
13. Ugliness vs. beauty	ديرالروايحللكلبالفايحكييشمهايرد dir ʔerwajaħ leka:lb ʔelfajaħ ki j∫amha jru:d **Transliteration:** Put the perfumes on the stinky dog and when it smells it, it denies it. **Possible translation:** Like perfuming the stinky dog, when it smells the good odor, it will give a cold shoulder to it. **Meaning:** This proverb is said to the person who is rude in nature, who cannot distinguish between antiques and trivial things. Therefore, the person who is used to ugly and stinky nature will not have the sense of tasting beautiful things.		-

The first category describing the inferiority and superiority of either a man over a woman or the ranking of genders in society can be seen in the proverb (اجت العنزة الثنيّة تعلّم أمها الرّعيّة) “*The young goat came to teach its mother how to graze*.” In this proverb, we can deduce how the scales are inverted, and a low-ranking animal aims to teach a superior one what to do. In the human context, it applies when the daughter surpasses her mother and wants to teach her how to do something. Therefore, the daughter, who is younger and inferior to her mother, attempts to teach her mother, who taught her the basics and the principles of life.

In describing the weakness of women, the Jordanians opt for the proverb (النّاقة ناقة ولو هدّرت), “*The camel is a camel even if it growls*” meaning that even if the female camel growls, it will still not make it a lion but remain a camel and weak. Correspondingly, a woman or a wife will always be perceived as weak and needing protection from her husband. This does not imply that the woman is ranked lower in this context but instead shows her need for a man to protect and support her. Furthermore, the proverb suggests that even when women shout or do anything to show their powers, they will still remain a woman, full of feelings and tenderness.

Proverb (16) (اللّي برضع لبن الحمارة بيصير مخّه حجارة) “*The one who drinks the milk of a donkey, his brain becomes a stone*” depicts that when the burro drinks its mother's milk, it will automatically inherit her stupidity and stubbornness. This applies to humans where a child breastfeeds from a mother who is stupid and stubborn automatically inherits these characteristics because the child assumes its intelligence from the mother. Therefore, being fed and raised by a stubborn mother will not result in an obedient child.

Snakes and scorpions are at the top of the list of animals in the proverbs that refer to the ill-nature inherent in some animals. Likening someone to a scorpion or a snake reveals that person's bad intentions or cunningness. Proverb (17) (حب العمّة للكنّة مثل لسع العقربا) “*The love of the mother-in-law to the daughter-in-law is like the bite of a scorpion*” expresses the dislike that the mother-in-law has for her son's bride. The relationship between a mother-in-law and the bride might appear in accord, but this would not be the case because the mother-in-law's love for her daughter-in-law is like the bite of a scorpion—poisonous and deadly.

The authority of a man over a woman or his wife and depicting women as a sex object is evident in Proverb (18): (فيه عى تر لا مرعاهن لكن نوق اعشق عشقت ان) “*If you love, love camels but do not graze in their pastures*.” When men in Jordanian society equate a beautiful woman to a camel, they describe her as the most beautiful among others. While urging men to fall in love with pretty women, this proverb cautions them against allowing women to imprint them with their nature. It calls for establishing the power of men over women and not allowing their sexual desires for women to guide their emotions.

Similarly, the proverb (جمل اسرق سرقت ان و نوق اعشق عشقت ان) “*If you love, love she-camels and if you steal, steal a camel*” urges men to marry a beautiful woman amongst other women. In Jordanian culture, when a woman is pretty and beautiful, men flirt with her, referring to her as a camel. Therefore, this proverb elucidates the beauty of a woman by equating it to a camel.

The adjective shrewd refers to someone who is skillful, cunning, or devious in nature, and its antonym is innocent. The proverb, (رة بالحا أمها و ة فار توخذ لا) “*Do not take the mouse when its mother is in the neighborhood*,” is used to defame the reputation of the mother-in-law, which is not always the case. Based on Khan's classification, shrewdness has to do with artfulness and cunning, and some people believe that a mother-in-law is a bad person who keeps scheming to get her son to divorce his wife. Conversely, it also refers to a mother influencing her daughter to be authoritative and bad with her husband.

Table 5 outlined how animal-related proverbs manifested and characterized women in Jordanian society. It is noticeable that women in Algerian and Jordanian cultures are sketched in a derogatory manner, unlike men, who are represented with power and authority over women. In this study, we found that louse, dog, cow, sheep, hen, donkey, snake, scorpion, and rat were used to describe women in a pejorative manner. For instance, the hen and cow animal terms are used as a metaphor for describing the weakness of women because both are inferior compared to their male counterparts in the proverbs. This is also reflected in the Algerian proverbs. These results are in line with Khan et al.'s (2017) study that investigated the portrayal of men and women in Urdu proverbs through animals. The horse and camel are the only two animal terms that have been used positively.

**Table 5:** Jordanian animal-related proverbs describing women.

**Categories**	**Women animal-related proverbs along with their translation**	**Positive meaning**	**Negative meaning**
14. Inferiority vs. superiority	اجتالعنزةالثنيّةتعلّمأمهاالرّعيّةʔidʒat ʔilʕanza ʔi θanija tʕalim u:mha ʔiraʕija **Transliteration:** The young goat came to teach its mother how to graze. **Possible translation:** The baby goat is now teaching its mother how to graze. **Meaning:** This proverb is said in cases where the little person who has no experience wants to be far above his/her master.		-
15. Weakness vs. strength	النّاقةناقةولوهدّرتʔinna:qa na:qa: walaw haddarat **Transliteration:** The camel is a camel even if it growls. **Possible translation:** The camel remains a camel even if it growls. **Meaning:** That is, the female remains a female, no matter how strong her personality is, she is limited in terms of her creation and composition, and she cannot be endowed with the characteristics of men. Because she was created to be a female, and she has her lofty message in life, and her great value in society.	+	
16. Stupidity vs. wisdom	اللّيبرضعلبنالحمارةبيصيرمخّهحجارةʔilli bjirdʕaʕ laban ħma:ra bi:sʕir muxxu ħdʒa:ra **Transliteration:** The one who drinks the milk of a donkey, his brain becomes a stone. **Possible translation:** He/she who is fed by the donkey's milk will become stubborn. **Meaning:** This means that when the burro drinks its mother's milk, it will automatically inherit her stupidity and stubbornness. That is to say, if a person has some features by birth and he grows up in a wrong manner, then it is not possible to change his behaviors. Just like a donkey that is known by its stupidity and obedience, it is not easy or even impossible to get used to being easygoing.		-
17. Ill-nature vs. good nature	حبالعمّةللكنّةمثللسعالعقرباħu:b lʕamma lalkanna miθl lasʕ ʔiʕaqraba **Transliteration:** The love of the mother-in-law to the daughter-in-law is like the bite of a scorpion. **Possible translation:** A mother-in-law's love for a daughter-in-law is like a scorpion's sting. **Meaning:** The boundless hatred between the mother-in-law and her daughter-in-law is like the scorpion's sting which is known by its poison and evil attitudes, therefore when a person is evil in their nature they are likened to a scorpion as to show that it is not possible to get rid of your bad and evil characteristics.		-
18. Sex object vs. authority	انعشقتاعشقنوقلكنمرعاهنلاترعىفيهʔin ʕa∫iqt ʔiʕ∫aq nu:q la:kin marʕahen la tirʕa: fi:h **Transliteration:** If you love, love camels but do not graze in their pastures. **Possible translation:** If you want to fall in love, fall in love with a camel, but do not graze in its pastures. **Meaning:** This saying urges men not to allow women to take control of them (do not acquire your wife's traits). This proverb like others belonging to this category depicts the authority of a man that he should show and practice in his first days of marriage; as to avoid his wife to take control over him or the like.	+	
19. Ugliness vs. beauty	انعشقتاعشقنوقوانسرقتاسرقجملʔin ʕa∫iqt ʔiʕ∫aq nu:q w ʔin ʔisraqt ʔisraq dʒamal **Transliteration:** If you love, love she-camels and if you steal, steal a camel. **Possible translation:** If you should fall in love, then fall in love with a she-camel, but if you want to settle down then take a camel. **Meaning:** This saying urges men to marry a beautiful lady amongst other ladies. In the Jordanian culture, when a woman is so pretty and beautiful then men mainly flirt her by saying she is camel. Therefore, this proverb shows the beauty of a woman by likening it to a camel.	+	
20. Shrewd vs. innocent	لاتوخذفارةوأمهابالحارة la tu:xeθ fa:ra w ʔimha bil ħa:ra **Transliteration**: Do not take the mouse when its mother is in the neighborhood. **Possible translation:** Do not take (as a wife) a rat if her mother is in the neighborhood. **Meaning:** This is mainly used to defame the bad reputation of the mother-in-law, which is not always the case. Following Khan et al.'s (2017) classification, shrewdness has to do with artful and cunning and it is believed by some people that the mother-in-law is a bad person who keeps doing tricks to get her son-in-law to divorce his wife, or the other way around, that is, pushing her daughter to be authoritative with her husband.		-

Table 6 contains the animal-related proverbs depicting men in Jordanian society. The proverbs in this subset included all the categories designed for this study, unlike the other subsets represented in other tables, which did not contain all eight categories (i.e., one or two categories were missing).

**Table 6:** Jordanian animal-related proverbs describing men.

**Categories**	**Men animal-related proverbs along with their translation**	**Positive meaning**	**Negative meaning**
21. Inferiority vs. superiority	الكلبكلبولولبسجلدأسدʔilka:lb ka:lb wa law libis dʒild ʔasad **Transliteration:** The dog is a dog even it wears the skin of a lion. **Possible translation**: The dog remains a dog even if it puts on a lion's skin. **Meaning**: The vile does not raise his value with his clothes. The dog as an animal is lowly ranked in the Great Chain of Being. Therefore, when it is compared to an animal higher than it, then this is an instance of inferiority and superiority.		-
22. Weakness vs. strength	يوميبركالجملتكثرالسكاكين ju:m jubruk ʔildʒamal tukθur ʔissakaki:n **Transliteration:** The day when the camel falls the knives will be too much. **Possible translation**: When the camel falls sick, ready knives become plentiful. **Meaning**: This is said in describing the strong who is weakened and afflicted by calamities from every side.	+	
23. Stupidity vs. wisdom	الفاراللّيشايفحالهبيكونمننصيبالبسʔilfa:r ʔilli ∫aji:f ħalu: biku:n min nasʕib ʔilbis **Transliteration:** The rat that thinks highly of itself is from the share of the cat. **Possible translation:** The arrogant rat is an easy meal for the cat. **Meaning:** This saying describes the mean and stupid person who likes to show off, so his arrogance and haughtiness will be the reason for his elimination.		-
24. Ill-nature vs. good nature	يوكلمعالذّيبويرعىمعالغنم Ju:kel maʕa ʔiððib wjarʕa maʕa ʔilɤanam **Transliteration:** He eats with the wolf and grazes with the sheep. **Possible translation:** He eats with the wolf and grazes with the sheep. **Meaning:** This proverb is used to describe a hypocritical person who reveals the opposite of what he hides, so his intentions cannot be predicted.		-
25. Sex object vs. authority	شووصّىعليك؟قال**:** وصاة الذّيب بالغنم ∫u: wasʕsʕa ʕalik qal wsʕat ʔiððib bil ɤanam **Transliteration:** What did he advise for you? The advice of a wolf over the sheep. **Possible translation:** They asked: What did he recommend to look after you? He replied: Like putting the sheep under the surveillance of the wolf. **Meaning:** Handing the power to the strong over the weak. This example shows the authority of the wolf over the sheep, which is a weak, powerless animal.		-
26. Ugliness vs. beauty	ياماخذالقردعلىمالهبيروحالمالوبيظلالقردعلىحاله ja maxiθ ʔilgird ʕala malu: biru:ħ ʔilmal w bidʕal ʔilgird ʕala ħalu: **Transliteration:** O you who takes the monkey for its money, the money goes and the monkey stays as it is. **Possible translation:** Whoever marries the monkey for his money, a day will come and he will be poor, and the monkey will remain a monkey. **Meaning:** This proverb serves to denounce marriage that is guided by a materialistic view. That is, marrying an ugly person just because of his wealth will make you regret 1 day after he becomes poor. In the classification suggested in the methodology, the monkey is mainly associated with ugliness.		-
27. Positive vs. negative	كلالجمالاتعاركالّاجملنابارك kul lidʒma:l ʔitʕa:rik ʔilla dʒamalna barik **Transliteration:** All the camels fight except for our camel, it sits. **Possible translation:** All the camels fight except for ours that is lying down. **Meaning:** This imagery depicts the laziness of some individuals who have nothing to do in their lives, they are passive. And as it has been said before, if there is a person among other groups with bad habits, he will affect all of them by time whether by his laziness or other characteristics.		-
28. Shrewd vs. innocent	ذنبالكلبأعوجلوحطّيتهبستّينقالب ðanab ʔilkalb ʔaʕwaj law ħatʕtʕitu bsittin qali:b **Transliteration:** The tail of the dog is crooked even if you put it in 60 models. **Possible translation:** The dog's tail remains curved and cannot be straightened even if it is molded in sixty models. **Meaning:** A leopard cannot change its spots. That is, if someone inherits bad habits, he will never be able to change his traits and vice versa.		-

The first proverb in the first category (الكلب كلب ولو لبس جلد أسد), “*The dog is a dog even it wears the skin of a lion*.” reveals the image of a dog, that despite putting on a lion's skin, will still remain a dog. It implies that a lower-ranking animal like a dog can never be comparable to or rise to the status of the lion. When transliterated to human beings, an ordinary man cannot rise to the position of a nobleman even if he adorns himself with ornaments because upbringing is more powerful than cosmetic changes.

As highlighted earlier, the camel symbolizes strength in Arabic culture, and its cow symbolizes beauty in Jordanian culture. Proverb (22) (يوم يبرك الجمل تكثر السكاكين) “*The day when the camel falls the knives will be too much*,” laments that when the camel is sick or weak, everyone would be ready to slaughter it. It implies that even the weak may want to challenge him when a strong man or a leader has lost everything and he is no longer powerful or in a powerful position. Subsequently, his enemies will conspire against him to ensure he no longer hinders them.

Some animals can try to be tricky, like the mouse in proverb (23) (من بيكون حاله شايف اللّي الفار البس نصيب) “*The rat that thinks highly of itself is from the share of the cat*.” An arrogant mouse that believes it can outsmart the cat will eventually end up as the cat's dinner. Likewise, for foolish individuals who think they can outsmart others, their overconfidence will cause their downfall.

Proverb (24) (يوكل مع الذّيب ويرعى مع الغنم) “*He eats with the wolf and grazes with the sheep*” warns of someone who will eat the sheep when he is with the wolves and pasture with it when he is with the sheep. It portrays a hypocritical person who hides his inherent sinful, evil, and hateful nature and displays the contrary.

To explain the authority of men over women, particularly the sexual desires men may have for women, the following proverb is used: (شو وصّى عليك؟ قال: وصاة الذّيب بالغنم) “*What did he advise for you? The advice of a wolf over the sheep*.” This proverb cautions against allowing the wolf to protect the sheep as it may lead to the wolf attacking the sheep. Likewise, a man with clear ill intentions should not be put in charge of someone else's security, particularly that of a woman.

In some cases, women overlook the physical appearance and other characteristics of a suitable husband if he has enough money. However, fortunes might change, and the man may lose all his money, and what he will be left with may not be desirable. This is explained in the following proverb (26): (حاله على القرد بيظل و المال ح بيرو ماله على القرد ماخذ يا) “*O you who takes the monkey for its money, the money goes and the monkey stays as it is*.” Simply put, it means that if one takes the monkey for his money, the money will someday disappear, and what will be left is a monkey. This proverb instructs that if a woman was to accept someone as undesirable as a monkey just because of his wealth, he may no longer remain rich, and the woman would be left with his ugliness, which she endured only because he was rich. Therefore, it calls for focusing not on the material aspects but on the appearance (values) of the person because it plays an essential role. The proverb has also been used to describe a rich but ugly man who gets married to a beautiful woman.

Proverb (27) describes people with either negative or positive features. It criticizes lazy people who have nothing to offer to society; they are passive and even a burden for others, unlike positive people who are characterized by their generosity, help, and the important role they play in society. The proverb (بارك جملنا الّا اتعارك الجمال كل) “*All the camels fight except for our camel, it sits*,” indicates the resentment when particular camels, which are known as strong and fast animals, especially in races, end up being idle. This imagery reflects the laziness of some individuals who do nothing in their lives and remain passive.

As previously discussed, an analysis of the category of shrewdness shows how some characteristics, such as cleverness, skillfulness, and other physical traits, are passed on from the mother or the father to their children. The proverb (قالب بستّين حطّيته لو أعوج الكلب ذنب) “*The tail of the dog is crooked even if you put it in 60 models*,” reflects the behavior and nature of some people who will never change. Just as the leopard cannot change its spots, a dog with a curved tail will never get straightened, even if 60 molds were used to fix it. This proverb is also used to disapprove of shrewd or ill-behaved people whose crookedness cannot be straightened.

## 5. Discussion

The study's first question was: What are the connotative meanings of animal-related proverbs used in the Algerian and Jordanian societies? The data recognized the wide range of connotative meanings that Algerian and Jordanian proverbs have relating to animals. In both languages, most of the meanings that characterized women were derogatory. They included traits such as frailty, ignorance, inferiority, cunning, and trickery. Although the proverbs describing men in both languages had the same characteristics, the image of women in Arab cultures was particularly subordinate and had a negative profile. In addition, men continued to be seen as having power, dominance, superiority, and strength over women.

Numerous animal names were employed in both cultures to represent the images of men and women, both negatively and positively, in response to the second research question: What are the animal terms used to conceptualize men and women, both negatively and positively, in the Algerian and Jordanian societies? Within the studied proverbs, the quantity of these using animal names was the first difference between the Arabic used in Algerian and Jordanian communities. In other words, Algerian Arabic proverbs used 23 animal names, while Jordanian Arabic used 19 animal terms. The second difference was that certain animal words were only used in Algerian or Jordanian Arabic. For instance, Algerian Arabic proverbs contained animal terms such as fly, fish, partridge, peacock, cat, deer, duck, bee, and cock. On the other hand, Jordanian Arabic used hyenas, scorpions, ants, and lice. Regarding the positive and negative connotations, pejorative connotations were more prevalent than positive ones; negative connotations were more common than the ones observed in the Algerian proverbs when defining both sexes.

The analysis revealed that in both languages, women had a more unfavorable profile than men. Such findings are consistent with some findings of earlier studies. For instance, the animal pair of cock-hen was comparable to that used in Jayawardena's (2015) study, which evaluated how women were depicted in Sinhala and French cultures and found that male domination prevailed. The cock-hen animal pair represented the wife's loyalty to her husband and his power over her, which was demonstrated by comparing the hen's actions to those of the cock. Similarly, the hen and cow metaphors—both weaker than their male counterparts in proverbs—were used to describe the fragility of women, which was also evident in the Algerian proverbs in our study. These results are in line with Khan et al.'s (2017) study on how men and women are portrayed in Urdu proverbs using various animals. The dog was described negatively in the Jordanian proverbs and was associated with men, consistent with the findings of Khan et al. (2017). The monkey was also symbolized as ugly and found mentioned in both studies. In their study, the snake was used to emphasize the negative representation of men and was more frequently associated with men. However, the Jordanian proverbs did not totally support this conclusion; only one proverb was used to describe men using a snake allegory in Jordanian Arabic, whereas women were seen as cunning, and the snake was more frequently used to indicate a woman with malicious intentions.

The Jordanian proverbs used animal terms like dog, wolf, crow, snake, monkey, burro, and hyena to relate to negative traits like cunningness, ugliness, and the abjection of some men. The camel, lion, bird, gazelle, and horse represented the positive traits associated with Jordanian men, such as beauty and bravery. The cock-hen animal pair utilized in the Algerian proverbs to represent the authority of men over women was replaced by the wolf-sheep animal pair in Jordanian proverbs to demonstrate this authority.

There are some similarities and differences between the findings of research that have investigated the use of animal names to describe gendered features in languages. In Mandarin Chinese, the dragon represents strength, power, and masculinity, according to Chiang and Knight (2010). No positive animal terms were found to be used to describe men or women in Vyzoviti and Michalopoulou's (2016) study of the Greek language. Similarly, the snake is frequently used to describe women in Mandarin Chinese, stressing their cunning and deceitful nature, according to Chiang and Knight (2010). Likewise, Vyzoviti and Michalopoulou's (2016) study discovered that the snake was employed to describe women's negative characteristics, including sly and cunning behavior. These results are consistent with the Jordanian proverbs that equate women's negative qualities more frequently with the snake than men's.

The conclusions of our study are consistent with those of Bousmah and Ventelou (2016), who looked at the use of animal metaphors in Algerian Arabic. Similar to the Jordanian proverbs, the study found that words like dog and wolf were frequently used to characterize undesirable attributes in men. In contrast, lions and horses were connected with favorable traits. Furthermore, we found that the cock-hen pair was commonly used to describe male authority over women in Algerian Arabic, while the wolf-sheep pair was used in Jordanian Arabic. This finding is in accordance with Bousmah and Ventelou's (2016) study.

Al-Harahsheh's (2020) findings on using various animal terms are remarkably similar to our study. For instance, both Algerian and Jordanian spoken Arabic dialects negatively refer to the animal name “donkey.” Al-Harahsheh's findings are in accordance with this; the animal is primarily connected with the masculine gender and is used to denote stupidity, dumbness, dunderheadedness, stubbornness, sluggishness, and vulgarity. In our study, the cow has a similar negative connotation and is typically connected with women. Similarly, Al-Harahsheh's findings are consistent with our study's analysis of the animal term monkey, which refers primarily to men and denotes ugliness. This phrase could occasionally be used to describe someone as being ugly.

The current study suggests that the peacock is appropriate when describing beauty. Al-Harahsheh, however, believes the peacock is used pejoratively when illustrating beauty and conceit. Similarly, according to Al-Harahsheh, the camel describes both sexes equally. However, in our study, how the camel is used to portray women differs from how it is used to show men with strength and physical might. This difference is primarily found in the Jordanian proverbs, though it is also seen in the Algerian proverbs, perhaps to a lesser extent. In our Jordanian data, proverbs with the word camel are used to describe strength and sometimes laziness in men, but in the case of women, such proverbs are used to describe beauty in women.

Both Algerian and Jordanian proverbs mention male and female gazelles. According to Al-Harahsheh, gazelles always imply a good meaning and allude to speed, beauty, gentleness, and agility. The scorpion and the snake are employed in Algerian and Jordanian Arabic to describe undesirable traits and malevolent intentions in women. These results are consistent with those of Al-Harahsheh. According to him, scorpions and snakes are used negatively to characterize someone's behavior; they signify hostility, cunningness, unreliability, and harm. The animal term sheep denoted naivete, benevolence, and frailty in our study. These behaviors were prevalent in Algerian and Jordanian Arabic and were consistent with Al-Harahsheh's findings. According to other experts, this term also describes people who are henpecked, subservient, followers, gullible, nice, and naive.

## 6. Conclusion

This study sought to uncover the connotative meanings depicted in animal-related proverbs used to describe the behavior of women and men in the Algerian and Jordanian societies. Thirty native speakers of Algerian and Jordanian Arabic who were enrolled at the University of Jordan received a questionnaire that contained 45 animal-related proverbs from Jordan and 46 from Algeria. The gender-based categories of analysis used by Khan et al. (2017) were adapted as a framework to examine the proverbs. The categories were inferiority, weakness, idiocy, bad nature, sex object, ugliness, positivity, and shrewdness. The investigation revealed that animal-related proverbs from Algeria and Jordan had a variety of connotative interpretations but predominately had negative connotations when describing women. This was true in both languages. Women were characterized by weakness, stupidity, inferiority, cunningness, and trickery. Although the proverbs describing men in both languages tended to share the same traits, women in Arab cultures were portrayed as particularly inferior and had a demeaning profile. On the other hand, men were described as possessing power, dominance, superiority, and strength over women. Positive connotations were also found where the proverbs referred to animals like gazelle, peacock, partridge, cat, and horse to describe women's attractiveness. Similarly, men's superior qualities—such as strength, courage, and superiority—were elaborated using animals like horses, camels, and lions. Our results were compared to other relevant studies, and similarities and differences were found both in terms of animals terms used and in terms of what or whom they described. In conclusion, culture and language play a crucial role in using animal metaphors to express gendered features and power relationships. The parallels and discrepancies between research show that animal terminology is culturally distinctive and that effective interpretation requires knowledge of the cultural context.

## Data availability statement

The original contributions presented in the study are included in the article/supplementary material, further inquiries can be directed to the corresponding author.

## Ethics statement

Ethical approval was not required for the study involving human participants in accordance with the local legislation and institutional requirements. Written informed consent to participate in this study was not required from the participants in accordance with the national legislation and the institutional requirements.

## Author contributions

ZM: writing the draft of original article, data collection, and conceptualization. NA: writing the draft of original article, supervision, methodology section, and discussion. MR: methodology section, review and editing, and writing part of original article draft. All authors contributed to the article and approved the submitted version.
